# The Value of Contrast-Enhanced Ultrasound (CEUS) in the Evaluation of Central Lung Cancer with Obstructive Atelectasis

**DOI:** 10.3390/diagnostics14101051

**Published:** 2024-05-18

**Authors:** Ehsan Safai Zadeh, Katharina Paulina Huber, Christian Görg, Helmut Prosch, Hajo Findeisen

**Affiliations:** 1 Department of Biomedical Imaging and Image-Guided Therapy, Medical University of Vienna, Vienna General Hospital, 1090 Vienna, Austria; ehsan.safaizadeh@meduniwien.ac.at; 2 Interdisciplinary Center of Ultrasound Diagnostics, Gastroenterology, Endocrinology, Metabolism and Clinical Infectiology, University Hospital Giessen and Marburg, Philipp University of Marburg, Baldingerstraße, 35037 Marburg, Germany; 3 Department of General Internal Medicine and Psychosomatics, University Hospital Heidelberg, 69120 Heidelberg, Germany; 4 Department for Internal Medicine, Red Cross Hospital Bremen, 28199 Bremen, Germany

**Keywords:** CEUS, ultrasound, central lung cancer, atelectasis, diagnosis

## Abstract

**Purpose**: To assess the diagnostic performance of contrast-enhanced ultrasound (CEUS) alongside contrast-enhanced computed tomography (CECT) in evaluating central lung cancer (CLC). **Materials and Methods**: From 2006 to 2022, 54 patients with CLC and obstructive atelectasis (OAT) underwent standardized examinations using CEUS in addition to CECT. The ability to differentiate CLC from atelectatic tissue in CECT and CEUS was categorized as distinguishable or indistinguishable. In CEUS, in distinguishable cases, the order of enhancement (time to enhancement) (OE; categorized as either an early pulmonary arterial [PA] pattern or a delayed bronchial arterial [BA] pattern of enhancement), the extent of enhancement (EE; marked or reduced), the homogeneity of enhancement (HE; homogeneous or inhomogeneous), and the decrease in enhancement (DE; rapid washout [<120 s] or late washout [≥120 s]) were evaluated. **Results**: The additional use of CEUS improved the diagnostic capability of CECT from 75.9% to 92.6% in differentiating a CLC from atelectatic tissue. The majority of CLC cases exhibited a BA pattern of enhancement (89.6%), an isoechoic reduced enhancement (91.7%), and a homogeneous enhancement (91.7%). Rapid DE was observed in 79.2% of cases. **Conclusions**: In cases of suspected CLC with obstructive atelectasis, the application of CEUS can be helpful in differentiating tumor from atelectatic tissue and in evaluating CLC.

## 1. Introduction

The obstruction of a bronchus’ air supply area and the absorption of alveolar air distal to the occlusion result in obstructive atelectasis (OAT) [1]. Central lung cancer (CLC) is one of the most common causes of obstruction in the bronchus and of the subsequent OAT [2,3]. In international guidelines, contrast-enhanced computed tomography (CECT) is recommended as the primary method of choice in the diagnosis and staging of lung cancer [4,5,6]. In CECT, the extent of the tumor, the presence of other suspected lesions or lymph nodes, and the best method of histologically confirming the tumor are evaluated [4]. However, demarcation of the central tumor or intra-atelectatic metastases from the atelectatic tissue is limited using CECT [2]. Previous studies have reported a sensitivity of only 42% for bolus-enhanced CT and 80% for dynamic-enhanced CT in differentiating tumor from atelectatic tissue [7,8], and therefore there remains a need to investigate new imaging procedures for achieving this differentiation [2]. In addition to CT, B-mode lung ultrasound (B-LUS) and contrast-enhanced ultrasound (CEUS) are widely used in clinical practice for the diagnosis and evaluation of pleural-based lung cancer and lung atelectasis [9,10,11,12,13,14,15,16,17,18]. Furthermore, in the case of CLC, sonographic visualization of a central cancer is feasible when a tumor-related atelectasis can be utilized as an “acoustic window” [9]. However, data on the diagnostic performance of B-LUS and CEUS in addition to CT in demarcating CLC from atelectasis, as well as data on the ultrasound patterns of CLC, are limited [19]. In cases of distinguishable tumors, a unique opportunity arises to study and describe the perfusion pattern of lung cancer in comparison with visualizable physiological lung tissue, such as atelectasis. The aims of the present study were to describe the ultrasound pattern and to evaluate the diagnostic performance of B-LUS and CEUS in addition to CECT for the demarcation of a CLC in patients with histologically confirmed central lung cancer and tumor-associated OAT. An additional aim of this study was to describe the ultrasound patterns of CLC in B-LUS and CEUS if differentiation between the tumor and atelectatic tissue is possible.

## 2. Materials and Methods

Between January 2006 and May 2022, a total of 54 patients with CLC and OAT were examined and standardized using B-LUS and CEUS by a German Society for Ultrasound in Medicine (DEGUM) Level-III qualified examiner with more than 35 years of experience in the field of thoracic sonography (C.G., internal medicine) at a university ultrasound center [20]. Central lung cancer was defined as a tumor located in the inner two-thirds of the hemithorax, according to the definition by the European society of thoracic surgery [21]. All the patients were referred to the ultrasound center for routine initial staging procedures for lung cancer. In all patients, according to the hospital’s internal standard operating procedures, a lung ultrasound, lymph node ultrasound, and abdominal ultrasound were performed systematically. Patients could only be included in the study if the subsequent atelectasis had contact with the pleural surface and the tumor was visible in the B-mode ultrasound. The examiner was blinded to the CT images. The inclusion criteria for the retrospective analysis were (1) histologic confirmation of CLC and (2) the availability of standard staging procedures with CECT. The ultrasound data were obtained according to hospital guidelines during general clinical procedures, and they were retrospectively evaluated. Informed consent was obtained from all the patients for the CEUS examination. This study was approved by the local ethics committee and performed in accordance with the revised Declaration of Helsinki.

### 2.1. Ultrasound Examination

The B-LUS examinations were performed using an ACUSON SEQUOIA 512 GI ultrasound machine (Siemens, Erlangen, Germany) and a 4C1 curved-array transducer with a frequency of 4 MHz. The ultrasound position was selected after evaluating the chest X-ray and based on the patients’ condition, including cooperation and anatomical considerations. The CEUS investigations were conducted with the same transducer in contrast-specific mode (1.5 MHz) with a low mechanical index (0.15–0.21) and in accordance with the European Federation of Societies for Ultrasound in Medicine and Biology (EFSUMB) guidelines [22]. A bolus injection of 2.4 mL of the contrast medium SonoVue^®^ (Bracco Imaging S.p.A., Milan, Italy) was performed via peripheral venous access. This was followed by 10 mL of NaCl 0.9%. Only a single injection was required for all study patients. No contrast-agent-related reactions were observed in the study participants. For the first 30 s, the perfusion patterns of the lesions were examined continuously and recorded as a video clip. Subsequently, several short examinations were performed at one-minute intervals up to 3 min, and the changes in the perfusion pattern were saved as images [23]. All ultrasound examinations were performed in the upright sitting position and horizontal to the ribs [23]. The B-LUS and CEUS data were evaluated retrospectively by two independent, experienced investigators (C.G., E.S.). In the event of discrepancies, the final decision was made by a third experienced investigator (H.F.). Cohen’s kappa statistics were applied to measure inter-rater reliability. The following B-LUS data and CEUS parameters were evaluated retrospectively [23].

### 2.2. B-LUS

1. The distinguishability of central tumor formation from atelectatic tissue was classified as distinguishable or indistinguishable. In distinguishable cases, the following additional CLC data were analyzed:

2. Echogenicity of the CLC compared with the OAT; 

3. Homogeneity of the CLC. 

### 2.3. CEUS

1. The distinguishability of central tumor formation from atelectatic tissue was classified as distinguishable or indistinguishable. In distinguishable cases, the following additional CLC data were analyzed:

2. The order of enhancement (OE) (time to enhancement) of the CLC and the OAT was determined and classified as an early pulmonary arterial (PA) pattern of enhancement (i.e., contrast enhancement of the lesion before the arrival of contrast agent in the thoracic wall) versus a delayed bronchial arterial (BA) pattern of enhancement (i.e., contrast enhancement of the lesion simultaneous with the arrival of contrast agent in the thoracic wall or parenchymal organs) [9,23]; 

3. The extent of enhancement (EE) at the peak of enhancement of the CLC compared with atelectatic tissue was categorized as reduced EE (hypoechoic) versus marked EE (isoechoic) [9,12,23];

4. The homogeneity of enhancement (HE) of the CLC and the OAT was classified in the arterial phase as homogeneous versus inhomogeneous enhancement [23,24,25,26]. A perfused lesion with coexisting nonperfused areas was defined as an inhomogeneous enhancement [23,24,25,26];

5. The decrease in enhancement (DE) of the CLC and the OAT was classified as a rapid washout (<120 s) or a late washout (≥120 s) [16,23]. A washout is defined as a parenchymal hypoenhancement compared to arterial peak enhancement.

## 3. Results

### 3.1. Demographic and Clinical Data

Of the 54 study patients, 14 (25.9%) were female and 40 (74.1%) were male. The mean age of the patients was 64.7 years (range 40–82 years). In all patients, the tumor was diagnosed as primary lung cancer following evaluation by the tumor board, which considered all imaging methods and histological findings. Histology confirmed that 20 of the 54 lesions (37.0%) were adenocarcinomas, 17 (31.5%) were squamous cell carcinomas, 14 (25.9%) were small cell lung carcinomas, 1 (1.9%) was a large cell carcinoma, 1 (1.9%) was a neuroendocrine carcinoma, and 1 (1.9%) was an undifferentiated carcinoma. The diagnosis of the 54 cases was confirmed by bronchoscopic biopsy in 40 cases (74.1%), via surgery in 5 cases (9.3%), by ultrasound-guided biopsy in 6 cases (11.1%), and by confirming distant metastases in 3 cases (5.6%) (2 in lymph nodes and 1 in the lumbar vertebrae). During the staging procedures, CECT enabled the differentiation of a central mass from atelectasis in 41 of 54 patients (76.0%). Differentiation between a central tumor and atelectasis could not be achieved in the remaining 13 patients (24.0%).

### 3.2. B-US

#### 3.2.1. Differentiation between the Tumor and Atelectatic Tissue on B-US

On B-LUS, differentiation between atelectasis and a central tumor was possible in 23/54 cases (42.6%). In the remaining 31/54 cases (57.4%), differentiation between CLC and OAT was not possible.

#### 3.2.2. Echogenicity of the Tumor

In distinguishable CLCs, compared with atelectasis, 19/23 cases (82.61%) were hypoechoic, 2/23 (8.70%) were isoechoic, and 2/23 (8.70%) were hyperechoic.

#### 3.2.3. Homogeneity of the Tumor

In B-LUS, 15/23 (65.2%) of the tumors were homogeneous, and 8/23 (34.78%) of the tumors were inhomogeneous.

The agreement between the examiners regarding the differentiation of CLCs and OATs was “good” (Cohen’s kappa = 0.78).

### 3.3. CEUS Data

#### 3.3.1. Differentiation between the Tumor and Atelectatic Tissue on CEUS

**Figure 1:**
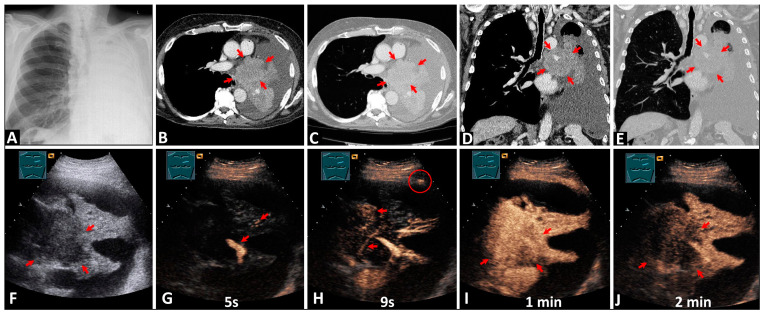
A 75-year-old patient with total opacity reduction in the left lung on chest X-ray (**A**) and suspected bronchogenic carcinoma. On computed tomography scans (**B**–**E**), a central tumor formation (arrows) with obstructive atelectasis is visible. On B-mode lung ultrasound (**F**), a central tumor (arrows) is distinguishable from the downstream atelectasis. On contrast-enhanced ultrasound, the atelectatic tissue shows inhomogeneous early pulmonary arterial enhancement (arrows) after 5 s, before the chest wall (**G**). The tumor tissue exhibits homogeneous delayed bronchial arterial enhancement (arrows) after 9 s, simultaneously with the chest wall (red circle), which is a sign of systemic vascularization (**H**). The tumor displays isoenhancement (arrows) compared with the atelectasis after 1 min (**I**), with rapid washout (arrows) after 2 min (**J**).

On CEUS, differentiation between atelectasis and a central tumor was possible in 48/54 cases (88.9%; Figure 1).

**Figure 2:**
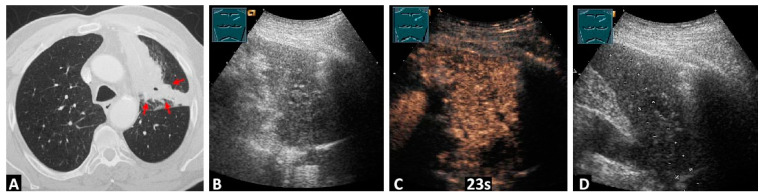
A 66-year-old male patient with left-sided thoracic pain and non-resolving pneumonia after 2 weeks of antibiotic therapy. Computed tomography scans (**A**) reveal a central tumor formation (arrows) with obstructive atelectasis. Due to significant secretion retention and respiratory instability, bronchoscopic histology collection could not be performed. In B-mode lung ultrasound (**B**), a central tumor is not distinguishable from the downstream atelectasis. In contrast-enhanced ultrasound (**C**), a central tumor can be differentiated from the atelectasis. The visualization of the tumor in contrast-enhanced ultrasound enabled a complication-free histologic confirmation of the central tumor formation through the subsequent atelectasis (**D**). The histology confirmed the diagnosis of squamous cell carcinoma of the left upper lobe.

In the remaining 6/54 cases (11.1%), differentiation between CLC and OAT could not be made. On CEUS, central tumors were identified in 25 cases that were not detectable by B-LUS (Figure 2, Video S1).

**Figure 3:**
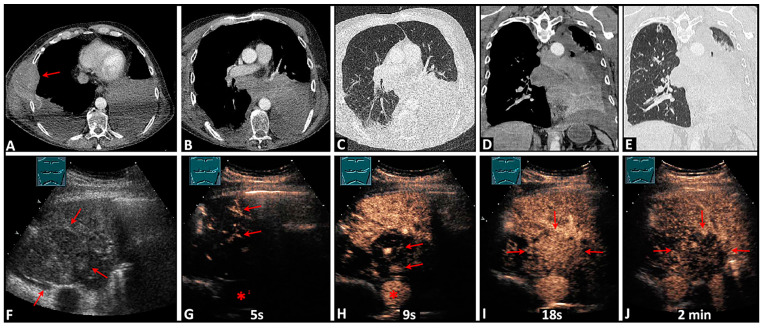
A 57-year-old patient with a bronchoscopically confirmed small cell bronchogenic carcinoma of the left lower lobe and contralateral rib and chest wall metastases (arrow) (**A**). On computed tomography scans (**B**–**E**), an obstructive atelectasis with pleural effusion is visible, but a tumor formation cannot be differentiated. On B-mode lung ultrasound (**F**), a central tumor is distinguishable from the downstream atelectasis (arrows). On contrast-enhanced ultrasound, the atelectatic tissue shows homogeneous early pulmonary arterial enhancement (arrows) after 5 s, before the aorta (*) (**G**). The tumor tissue exhibits homogeneous delayed bronchial arterial enhancement (arrows) after 9 s, simultaneously with the aorta (*), which is a sign of systemic vascularization (**H**). The tumor displays isoenhancement (arrows) compared with the atelectasis after 18 s (**I**), with rapid washout (arrows) after 2 min (**J**).

Additionally, on CEUS, central tumors were revealed in 10 cases that were not detectable by CT (Figure 3, Video S2).

**Figure 4:**
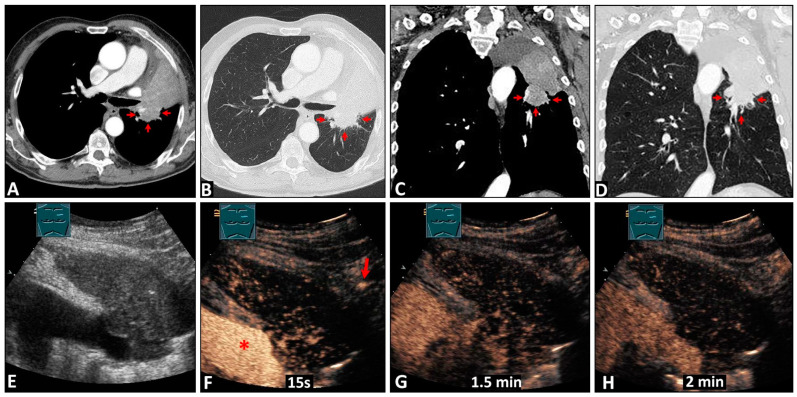
A 75-year-old patient with a bronchoscopically confirmed squamous cell carcinoma of the left upper lobe. On computed tomography scans (**A**–**D**), a central tumor formation (arrows) with obstructive atelectasis is visible. On B-mode lung ultrasound (**E**), a central tumor is not distinguishable from the atelectatic tissue. On contrast-enhanced ultrasound, the atelectatic tissue shows homogeneous delayed bronchial arterial enhancement simultaneously with the chest wall (arrow) after the enhancement of the pulmonary artery (*), which is a sign of systemic vascularization (**F**). After 1.5 min, a tumor formation is still not distinguishable (**G**). The atelectasis shows rapid washout after 2 min (**H**).

Furthermore, in three cases, tumors could not be detected by CEUS but were detectable by CT (Figure 4). 

The diagnostic performance of CEUS compared with B-LUS and CT is presented in Table 1.

The agreement between the examiners regarding the differentiation of CLC and OAT was “good” (Cohen’s kappa = 0.78). In two cases, the tumor and atelectasis were distinguishable by the first examiner but indistinguishable to the second examiner. The final decision made by the third examiner was that the tumor and atelectasis were indistinguishable. Order of enhancement

Regarding OE in distinguishable CLC, 43/48 lesions (89.6%) revealed a delayed enhancement due to BA perfusion, and 5/48 lesions (10.4%) showed an early enhancement due to PA perfusion. The OAT showed an early PA enhancement due to PA perfusion in 45/48 cases (93.75%) and a delayed enhancement due to BA perfusion in 3/48 cases (6.25%). In 8/48 cases (16.67%), the tumor and the atelectasis had the same OE, with 3 cases showing a BA pattern of enhancement and 5 cases showing a PA pattern of enhancement. In the remaining 40/48 cases (83.3%), the CLC and OAT had different OEs (the tumor having a BA pattern of enhancement and atelectasis having a PA enhancement).

In 6/54 cases (11.1%) with an indistinguishable central tumor, the atelectasis showed a BA enhancement in 5 cases (83.3%), and a PA enhancement was observed in 1 case (16.7%). 

#### 3.3.2. Extent of Enhancement

Regarding EE, compared with the atelectasis, the tumors exhibited hypoenhancement in 44/48 cases (91.7%) and isoenhancement in 4/48 cases (8.3%).

#### 3.3.3. Homogeneity of Enhancement

Regarding HE, 44/48 CLCs (91.7%) showed homogeneous enhancement, and 4/48 CLCs (8.3%) showed inhomogeneous enhancement. The OAT exhibited homogeneous enhancement in 34/48 cases (70.8%) and inhomogeneous enhancement in 14/48 cases (29.2%). In 6/54 cases (11.1%) with an indistinguishable central tumor, the atelectasis showed homogeneous enhancement in 5 cases (83.3%), and inhomogeneous enhancement was observed in 1 case (16.7%).

#### 3.3.4. Decrease in Enhancement

Regarding DE, 46 CLCs showed a rapid washout (<120 s), and 2 CLCs showed a late washout (>120 s). The OAT exhibited a rapid washout (<120 s) in 10 cases and a late washout in 38 cases. In 9/48 cases (18.8%), the CLC and OAT showed the same washout pattern (both rapid washout). In 38/48 cases (79.2%), the CLC showed a rapid washout, and the OAT showed a late washout; in 1/48 cases (2.1%), the CLC showed a late washout, and the OAT showed a rapid washout.

In 6/54 cases (11.1%) with an indistinguishable central tumor, the atelectasis showed a rapid washout in 5 cases (83.3%), and a late washout was observed in 1 case (16.7%).

## 4. Discussion

In accordance with international guidelines for the staging and diagnosis of bronchogenic carcinoma, CECT is the diagnostic method of choice in patients with known or suspected lung cancer [27,28]. However, the use of ultrasound in addition to CT could be helpful in staging and in the acquisition of histologic samples [29,30,31,32,33]. The use of ultrasound-guided thoracentesis and endobronchial ultrasound for staging and histologic sampling of lymph nodes suspected of malignancy has been established in the guidelines for lung cancer [30]. Furthermore, it is already known that the use of thoracic ultrasound, complementary to CT, can improve the detection rate of supraclavicular lymph node invasion by approximately 20% and peritumoral atelectasis by approximately 26% [31]. In this standardized study, we investigated the value of ultrasound in the diagnosis and staging of central lung cancer. The results demonstrated that the diagnostic accuracy of CEUS in differentiating central tumors from atelectatic tissue is significantly superior at 88.9% compared with conventional B-LUS at 42.6%. Another important finding of this study was the demonstration that the use of CEUS in addition to CECT can be helpful in differentiating CLCs from atelectatic lung tissue. On CEUS, a central tumor could be differentiated from atelectatic tissue in 10 additional cases, thereby increasing the diagnostic performance of CECT from 75.9% to 92.6% with the additional use of CEUS.

The reason for this improvement could be attributed to the ability of CEUS to differentiate between chronic pathologies and normal lung tissue. It is known that the pulmonary artery has limited and the bronchial artery has pronounced neoangiogenesis capabilities [34,35]. Healthy lung tissue, including atelectatic lung tissue, and acute processes, such as acute pneumonia, are supplied by the pulmonary artery [14,23,36]. Conversely, chronic processes with neoangiogenesis, such as chronic inflammation or lung cancer, are predominantly supplied by BA perfusion [10,23,35]. Differentiation between the PA pattern of enhancement and the BA pattern of enhancement is possible with CEUS [37]. The contrast agent first reaches the right heart and pulmonary arteries and then the lung tissue supplied by the pulmonary artery (atelectatic lung tissue). Subsequently, the contrast agent reaches the left atrium and left ventricle, and then passes through the aorta to the systemic vasculature, including lung neoplasms supplied by the bronchial artery [37]. Therefore, CEUS enables differentiation between intact lung tissue with PA supply and chronic processes with neoangiogenesis, resulting in consecutive systemic BA perfusion [37]. However, for differentiating between these two processes, the time until contrast enhancement should not be used; instead, an in vivo reference with systemic vascularization should be utilized [37]. The time in seconds until contrast enhancement depends on many factors, such as cardiac function and venous access route (peripheral or central) [37]. Quarato et al., in a prospective study, showed that the strict measurement of the arrival time of the contrast agent (>10 s or <10 s) is not significantly different between benign and malignant processes [38]. Therefore, it may be more appropriate to use the term “order of enhancement” instead of “time to enhancement”. However, the term “time to enhancement” proposed by WFUMB is also not incorrect, as it does not refer to an exact time but rather to a time difference in contrast enhancement between consolidations with the PA pattern of enhancement and those with the BA pattern of enhancement [37]. Furthermore, it should be clear that a BA pattern is not indicative of malignancy but points to a chronic process with neoangiogenesis. It can occur not only in malignant chronic processes, like bronchial carcinoma or metastases, but also in chronic benign processes, like granulomatous inflammation and organized pneumonia [23,39]. Furthermore, chronic processes, probably due to pathological shunt formations [40], show a rapid decrease in enhancement [15,16]. In the current study, CLC and OAT showed a different OE in 83.3% of cases and a different DE in 81.2% of cases. Thus, during the examination time, differentiation between chronic processes (CLC) and normal lung tissue (atelectasis) was possible due to these differing perfusion patterns. However, differentiation between CLC and OAT is difficult when there is chronic atelectasis with a BA perfusion pattern of enhancement. In these cases, differentiation between tumor and atelectatic tissue may not be possible due to the similar perfusion of both chronic tissues. In this study, five of six cases of indistinguishable tumors had OAT with a BA pattern of enhancement. This study also investigated the pattern of central lung cancer on B-LUS and CEUS. On B-LUS, CLC lesions were predominantly hypoechoic in 82.6% of cases, thus displaying a pattern similar to that of pneumonia or chronic inflammation [23,41]. Furthermore, 65.2% of the tumors were homogeneous and 34.8% of the tumors were inhomogeneous on B-LUS. This finding indicates that CLC exhibits a heterogeneous pattern on B-LUS, and the B-LUS characteristics alone are not a suitable method for evaluating the malignancy of lesions suspected to be bronchogenic carcinomas. On CEUS, CLCs predominantly (89.6%) exhibited a BA perfusion pattern of enhancement with rapid washout. These findings align with the perfusion pattern of peripheral lung cancer on CEUS [10]. CLC showed inhomogeneous enhancement in only 8.3% of cases, which was significantly less than peripheral lung carcinomas. Peripheral lung carcinomas were more than 70% inhomogeneous in a previous study [10]. This could indicate better perfusion of CLC and, thus, smaller necrotic areas compared with peripheral lung carcinomas. Regarding the enhancement pattern, 91.7% of the CLCs showed reduced enhancement, a proportion significantly higher than that observed in peripheral lung cancer (40.5%) [10]. The reason might be the use of atelectatic tissue as a reference lesion for evaluating CLC: atelectasis shows marked PA enhancement, making the tumors appear more frequently as hypoenhanced in comparison [10]. Furthermore, CLC predominantly (79.2%) showed a rapid washout (<120 s), in line with findings described in the literature for neoplastic lesions [15,16]. The results of this study are relevant because they correlate with histopathologic examinations and show the perfusion pattern of normal lung tissue (as atelectasis) adjacent to a chronic process with neoangiogenesis, such as bronchial carcinoma, and can support earlier studies with the same presentation of normal lung tissue and tumor tissue [23,34,35] (Table 2). However, it must also be emphasized that there are exceptions, such as bronchial carcinomas with a lepidic growth pattern and malignant lymphomas (Table 2). In the aforementioned entities, the vascularization, likely due to the intact lung structure, may continue to be supplied by the pulmonary artery, and only in later stages, when the lung architecture is destroyed, does a shift from the PA pattern of enhancement to the BA pattern of enhancement occur. Moreover, in cases of obstructive atelectasis, this study identified variable patterns. While atelectatic lung tissue in this study predominantly exhibited a prolonged, homogeneous PA enhancement pattern, consistent with previous studies [14], variable perfusion patterns were observed in some cases within this study. In the case of chronic atelectatic lung tissue, a shift from PA supply to BA supply can occur. In this study, 14.8% of cases showed a BA pattern of enhancement, 27.8% displayed inhomogeneous enhancement, and 27.8% of cases demonstrated a rapid DE (Table 2). These perfusion patterns are consistent with chronic atelectasis and suggest neoangiogenesis with destruction of the normal lung architecture.

The findings of the present study are particularly relevant for the histologic confirmation of central tumors accompanied by subsequent atelectasis, especially when bronchoscopic sampling is not feasible and an ultrasound-guided biopsy is necessary. Lei et al. have already shown that the use of CEUS can be beneficial in the histologic evaluation of central tumor formations [19]. A puncture success rate of 98% for CEUS-guided biopsies has been described [19]. Moreover, the only complication observed was hemoptysis, with no instances of pneumothorax detected. For the CT-guided percutaneous transthoracic lung biopsy, the overall pooled incidence of pneumothorax is approximately 26% [32]. However, it is important to emphasize that ultrasound can target only lesions that are either caused by atelectasis or are in direct contact with the pleural surface, hence the low probability of pneumothorax. In contrast, CT can also target tumors located centrally within the lung and surrounded by air-filled lung tissue. Therefore, pneumothorax in these cases during CT-guided procedures is not unavoidable. Nonetheless, such cases cannot be visualized or targeted with ultrasound due to total reflection at air interfaces. For lesions visible on ultrasound, however, the needle can be monitored throughout the entire procedure, which is not possible with CT. Furthermore, the contrast agent in CEUS is strictly intravascular, enabling a more precise differentiation between vital and nonvital tissue [33]. Another advantage could be the improved assessment of the tumor’s extent if targeted radiotherapy is decided upon [2].

Furthermore, these results may contribute to a better understanding of the fundamentals of contrast-enhanced ultrasound for lung lesions, aiding in the development and application of this radiation-free and cost-effective method.

## 5. Limitations of the Study

There were some limitations to this study. These include the general limitations of lung ultrasound examinations, which are characterized by high interobserver and interequipment variability [43]. Furthermore, the study was conducted solely on patients who were referred to the interdisciplinary center of ultrasound diagnostics with suspected or confirmed lung cancer; therefore, selection bias cannot be ruled out. Furthermore, in this study it was not possible to perform a histological confirmation of obstructive atelectasis and the demarcated tumor in CEUS due to ethical reasons, therefore the differentiation between tumor and atelectasis remains a “presumed” differentiation. Another limitation of our study is the semi-quantitative classification of the ultrasound data, which may allow greater interpretive flexibility than a quantitative approach would. However, the inter-rater observer variability for the ultrasound findings demonstrated “good” agreement. Due to the retrospective nature of this study and the relatively small number of subjects (N = 54), further prospective multicentric studies are needed to validate our findings.

## 6. Conclusions

In summary, the results of this study indicate that CEUS may be useful in evaluating CLC. It has been previously described that the use of CEUS in addition to B-LUS is advantageous for the histologic sampling of central tumors with OAT [44]. However, it must be emphasized that a CLC can be visualized on ultrasound only if there is subsequent atelectasis present. Furthermore, CT offers a better overview compared with ultrasound, and, in ultrasound, only approximately 70% of the pleural surface can be examined [45]. The superior overview provided by CT enables the visualization of additional potential pathologies, which is indispensable for staging. Therefore, CT remains the gold standard, and B-LUS and CEUS remain only as additional methods to CT. CEUS in addition to CT can be employed in unclear cases or for histologic confirmation of central tumors. To assess the clinical relevance of these data and to validate our findings, further prospective multicentric studies are needed.

## Figures and Tables

**Table 1:** Diagnostic performance of CEUS compared with B-LUS and CECT.

Imaging Modality	Demarcation of Tumor from Atelectasis
B-LUS	23/54 (42.6%)
CECT	41/54 (75.9%)
CEUS	48/54 (88.9%)
CEUS and CECT	50/54 (92.6%)

B-LUS: B-mode lung ultrasound; CECT: contrast-enhanced computed tomography; CEUS: contrast-enhanced ultrasound.

**Table 2:** CEUS perfusion patterns of pulmonary inflammatory and neoplastic lesions.

Underlying Disease	Central Lung Cancer	Peripheral Lung Cancer	Lung Lymphoma	Obstructive Atelectasis	Acute Pneumonia	Granulomatous Disease	Organized Pneumonia
**Author**	Present study	Findeisen et al. [10]	Trenker et al. [42]	Present study	Linde et al. [36]	Safai Zadeh et al. [23]	Safai Zadeh et al. [39]
**No. of cases**	48	89	6	54	50	10	38
**Year**	2024	2019	2018	2024	2012	2021	2021
**Pattern of enhancement on CEUS**							
**OE (TE): PA** **BA**	10.4% 89.6%	28.1% 71.9%	83.3% 16.7%	85.2% 14.8%	92.0% 8.0%	0% 100%	28.9% 71.1%
**EE: Marked** **Reduced**	8.3% 91.7%	59.5% 40.5%	100% 0.0%	n.a.	74.0% 26.0%	0% 100%	76.3% 23.7%
**HE: Hom** **Inhom**	91.7% 8.3%	23.6% 76.4%	66.7% 33.3%	72.2% 27.8%	78.0% 22.0%	0% 100%	18.4% 81.6%
**DE: Rapid** **Late**	79.2% 20.8%	n.a.	50.0% 50.0%	27.8% 72.2%	n.a.	100% 0%	50.0% 50.0%

BA: bronchial arterial; CEUS: contrast-enhanced ultrasound; DE: decrease in enhancement; EE: extent of enhancement; HE: homogeneity of enhancement; Hom: homogeneous; Inhom: inhomogeneous; n.a.: not analyzed; PA: pulmonary arterial; OE: order of enhancement;TE: time to enhancement.

## Data Availability

The data are not publicly available due to privacy and ethical restrictions.

## Supplementary Materials

The following supporting information can be downloaded at: https://www.mdpi.com/article/10.3390/diagnostics14101051/s1.
